# Dynamics of protein secretion during adipocyte differentiation

**DOI:** 10.1002/2211-5463.12091

**Published:** 2016-06-15

**Authors:** Koichi Ojima, Mika Oe, Ikuyo Nakajima, Susumu Muroya, Takanori Nishimura

**Affiliations:** ^1^ Animal Products Research DivisionNARO, Institute of Livestock and Grassland ScienceTsukubaIbarakiJapan; ^2^ Research Faculty of AgricultureHokkaido UniversitySapporoHokkaidoJapan

**Keywords:** adipocyte, adipokine, extracellular matrix, proteomics, secreted proteins

## Abstract

The major functions of adipocytes include both lipid storage and the production of secretory factors. However, the type of proteins released from mouse 3T3‐L1 cells during adipocyte differentiation remains poorly understood. We examined the dynamics of secreted proteins during adipocyte differentiation using mass spectrometry (MS) combined with an iTRAQ ^®^ labeling method that enables the simultaneous analysis of relative protein expression levels. A total of 215 proteins were identified and quantified from approximately 10 000 MS/MS spectra. Of these, approximately 38% were categorized as secreted proteins based on gene ontology classification. Adipokine secretion levels were increased with the progression of differentiation. By contrast, levels of fibril collagen components, such as subunits of type I and III collagens, were decreased during differentiation. Basement membrane components attained their peak levels at day 4 when small lipid droplets accumulated in differentiated 3T3‐L1 cells. Simultaneously, peak levels of collagen microfibril components that comprise type V and VI collagen subunits were also observed. Our data demonstrated that extracellular matrix components were predominantly released during the early and middle stages of adipocyte differentiation, with a subsequent increase in the secretion of adipokines. This suggests that 3T3‐L1 cells secrete adipokines after their ECM is constructed during adipocyte differentiation.

**Abbreviations**
CM: conditioned medium
DM: differentiation medium
DMEM: Dulbecco's Modified Eagle Medium
ECM: extracellular matrix
FBS: fetal bovine serum
GM: growth medium
iTRAQ^®^: isobaric tags for relative and absolute quantitation
ITS: insulin, transferrin, and selenous acid
MS: mass spectrometry

Adipose tissue is a specific organ that plays a key role in lipid storage. In addition to its classical function as energy storage, adipose tissue produces a wide variety of secretory factors known as adipokines. Adipokines modulate the metabolic process in fat and other tissues 1, 2. The first identified adipokine, leptin, plays an important role in body weight control 3. Adiponectin is exclusively produced by adipose tissue to control metabolic processes via the regulation of glucose levels and fatty acid breakdown 4. More than 40 types of adipokines have been identified with endocrine, paracrine, and autocrine functions 5. This indicates that adipose tissue acts as an endocrine organ that secretes adipokines.

In adipose tissue, adipocytes are embedded in the extracellular matrix (ECM) network that predominantly consists of collagen and proteoglycans 6. Collagen fibrils are primarily comprised of collagen type I and type III fibrils, and provide fundamental ECM architecture to maintain structural integrity in adipose tissue. Endomysial collagen deposition is indispensable for the growth and differentiation of adipocytes *in vivo* 7. Each adipocyte is surrounded by the basement membrane that includes type IV collagen, laminin, and heparan sulfate proteoglycan 8. Collagen type V and type VI are secreted from cultured adipocytes to facilitate triglyceride accumulation during differentiation *in vitro* 9. These studies show that adipocytes produce various types of ECM components as releasing factors to provide mechanical support to adipocytes and to regulate adipogenesis.

The cultured mouse adipogenic cell line 3T3‐L1 alters its cell status, namely, proliferation and differentiation, depending on culture conditions 10. Following induction of adipocyte differentiation, adipocytes increasingly accumulate intracellular lipid droplets, leading to morphological alterations where spindle‐shaped cell becomes a round cell namely, adipocyte differentiation dynamically changes the intracellular metabolic system and cell morphology. We hypothesize that differentiating adipocytes release distinct secretory factors at different stages of adipocyte differentiation as the dynamic morphological and metabolic changes occur during adipocyte differentiation. To date, microarray and quantitative PCR approaches have been extensively applied to reveal changes in gene expression during adipocyte differentiation 11, 12, 13, 14. Nonetheless, there are a limited number of studies that have evaluated alterations of proteins released from differentiating adipocytes due to technical difficulties with the application of mass spectrometry (MS) analyses 15, 16, 17, 18. In this study, we used a proteomic approach to assess our hypothesis. Adipocyte‐derived proteins released at three stages of adipocyte differentiation were collected and labeled with iTRAQ^®^, enabling the determination of multiplexed relative quantification of proteins using isobaric tags 19, 20. MALDI‐TOF MS and subsequent tandem mass spectrometry (MS/MS) were applied to quantitatively identify proteins. An adipocyte‐derived releasing protein profile was constructed during adipocyte differentiation.

## Materials and methods

### Cell culture

The mouse embryo 3T3‐L1 cell line was purchased from the American Type Culture Collection. The 3T3‐L1 cells were cultured in growth medium [GM; 10% FBS in low‐glucose Dulbecco's Modified Eagle Medium (DMEM; Life Technologies, Grand Island, NY, USA)] for 3 days for cell proliferation. To induce adipocyte differentiation, mouse 3T3‐L1 cells were cultured in DM1 [10% FBS, 0.5 mm of 1‐methyl‐3‐isobutylxanthine (Wako Pure Chemical Industries, Ltd., Osaka, Japan) and 0.25 μm dexamethasone (Life Technologies) in high‐glucose DMEM] for 2 days. Cells were kept in DM2 [10% FBS in high‐glucose DMEM containing 5 μg·mL^−1^ insulin (Life Technologies)] and the medium was changed every other day 21. All media were supplemented with 100 U·mL^−1^ penicillin and 0.1 mg·mL^−1^ streptomycin (Life Technologies). FBS was filtered with a 0.45 μm filter to remove debris before use.

### Collection of medium

**Figure 1:**
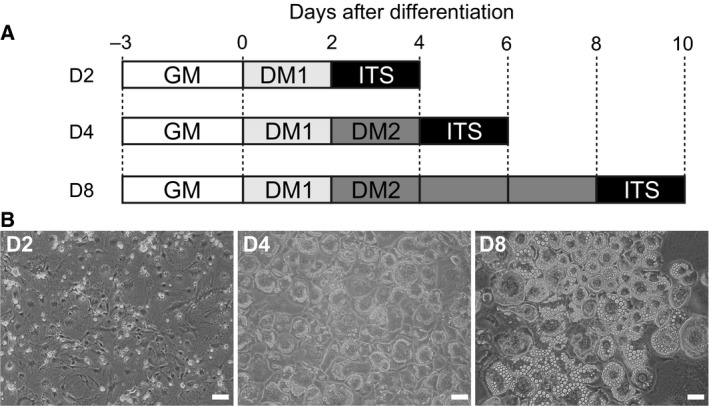
Adipocyte differentiation of 3T3‐L1 cells in the presence of ITS. (A) Scheme depicting 3T3‐L1 cell culture. Cells were kept in growth medium (GM, 10% FBS in low‐glucose DMEM) for 3 days for cell proliferation, followed by replacement of GM with DM1 (10% FBS, 0.5 mm 1‐methyl‐3‐isobutylxanthine, and 0.25 μm dexamethasone in high‐glucose DMEM) for 2 days to initiate adipocyte differentiation. To further induce adipocyte differentiation, cells were cultured in DM2 (10% FBS in high‐glucose DMEM containing 5 μg·mL^−1^ insulin) for 2 or 6 days. The culture media were changed to ITS (insulin, transferrin, and selenous acid) media on day 2 (D2), day 4 (D4), and day 8 (D8) following differentiation. CM was collected 2 days after feeding with ITS media. (B) Light microscopy‐based images of 3T3‐L1 cells cultured in ITS medium during adipocyte differentiation. Images were taken prior to the collection of CM. Small lipid droplets were observed on day 4. Mature adipocytes with a large number of lipid droplets were frequently found on day 8. Scale bars represent 50 μm.

Two days prior to the collection of conditioned medium (CM), the medium was changed from DM1 or DM2 to serum‐free media to avoid FBS contamination. At subsequent replacement of the media, cells were rinsed with 37 °C PBS at least thrice to eliminate serum contamination. Cells were then rinsed with high‐glucose DMEM that was retained in a cell incubator to adjust the pH and temperature (37 °C) at least 1 day prior to use. Cells were incubated with 6 mL of DMEM with ITS^™^ premix supplement (Becton Dickinson, Bedford, MA, USA) per 90 mm dish for 2 days. The ITS^™^ premix supplement contained insulin, transferrin, and sodium selenite, and was used as a substitution to FBS (Fig. 1). CM was collected, centrifuged, and filtered using 0.45 μm filters (Sartorius, Goettingen, Germany) to ensure removal of any debris. At each stage of cell culture, 120–240 mL of CM or 20–40 × 90 mm dishes for each specimen was collected. The CM was fractionated and concentrated using a series of spin columns with a cut‐off of 3, 50, and 100 kDa (Millipore, Billerica, MA, USA). The CM was fractionated into two classes to eliminate contamination of transferrin (76 kDa) that was present in the ITS^™^ premix supplement. One high molecular weight protein fraction was passed through 100 kDa cut‐off spin columns. The other fraction contained low molecular weight proteins that were passed through 50 kDa cut‐off spin columns but not 3 kDa cut‐off spin columns. Protein concentrations were determined using the Bradford protein assay (Bio‐Rad, Hercules, CA, USA). Equivalent amounts of secreted proteins in the CM were subjected to SDS/PAGE and the gel was stained with SYPRO^®^ Ruby (Bio‐Rad). SYPRO^®^ Ruby fluorescent signals were scanned using the Ettan DIGE imaging system (GE Healthcare, Buckinghamshire, UK).

### MS sample preparation and analysis

Sample preparation of CM and iTRAQ^®^ labeling for MS were performed as previously described 22, 23. Due to low specimen yields, a single iTRAQ labeling and MS run was performed. Briefly, 20 μg of the CM proteins at each time point were dissolved in 20 μL of 0.5 m triethylammonium bicarbonate containing 0.1% Rapigest (Waters, Milford, MA, USA). Each specimen was reduced with tris (2‐carboxyethyl) phosphine (T‐cep; ThermoScientific, Waltham, MA, USA), sulfenylated with methyl methanethiosulfonate (MMTS; Thermo Scientific), and treated with trypsin (Promega, Madison, WI, USA) for 16 h at 37 °C. An enzyme‐to‐substrate ratio was 1 : 10. Samples were labeled with iTRAQ^®^ 8‐plex reagent (ABSciex, Framingham, MA, USA) according to the manufacturer's instructions. Specimens were passed through 50 kDa cut‐off spin columns prepared from cultured cells at D2, D4, and D8, and were labeled with iTRAQ^®^ 113, 114, and 115, respectively. Specimens that did not pass through 100 kDa cut‐off spin columns prepared from cultured cells at D2, D4, and D8 were labeled with iTRAQ^®^ 116, 117, and 118, respectively. Specimens labeled with iTRAQ^®^ were mixed and multi‐step peptide fractionation was performed using a strong cation exchange column (ABSciex). A total of seven fractions were eluted with 20, 50, 75, 100, 150, 200, and 350 mm KCl, respectively. Each specimen was desalted using a Sep‐Pak cartridge (Waters) and concentrated using a centrifugal vacuum concentrator.

Peptide fractionation was performed using a nano‐flow LC system (Chorus 220; CS Analytics, Hants, UK) equipped with a MALDI‐plate spotter (AMR, Tokyo, Japan). Peptides trapped on a trapping column (0.3 × 5 mm, 5 μm, 12 nm, L‐column ODS; CERI, Tokyo, Japan) were separated onto an analytical column (0.075 × 50 mm) packed with Magic C18AQ resin (3 μm, 10 nm particles; Michrom Bioresources, Auburn, CA, USA). Peptides were eluted using a gradient of 5–40% solvent B in solvent A for 60 min (solvent A, 1.2% acetonitrile and 0.1% TFA; solvent B, 81.2% acetonitrile and 0.1% TFA), 40–100% solvent B in solvent A for 20 min, and 100% solvent B for 10 min. The column effluent was mixed with the MALDI matrix solution [2 mg·mL^−1^ α‐cyano‐4‐hydroxycinnamic acid (Shimadzu, Kyoto, Japan) dissolved in 70% acetonitrile containing 0.1% TFA] at a flow rate of 1.8 μL·min^−1^ at the outlet and was spotted directly onto ABI 4800 MALDI plates (ABSciex).

Spotted fractions corresponding to seven gradient segments in SCX chromatography (192 × 7 = 1344 spots) were analyzed using a mass spectrometer (ABSciex 4800 plus MALDI‐TOF/TOF Analyzer). Proteins were identified using an in‐house Mascot search engine (ver. 2.4.0.; Matrix Science, London, UK) and the NCBI database (20 571 509 sequences; 7 061 663 751 residues; 20120927). Contaminations were identified by the contaminants database which contains so‐called contaminated‐proteins such as serum proteins, trypsin, keratin, and so on (252 sequences; 128 178 residues; 20120927). Mascot search criteria for the NCBI database were as follows: Taxonomy, ‘Rodentia (Rodents)’; Enzyme, ‘trypsin’; Fixed modifications, ‘iTRAQ8plex (N‐term)’ and ‘iTRAQ8plex (K)’; Variable modifications, ‘Deamidated (NQ)’, ‘Oxidation (M)’, ‘MMTS (C)’, and ‘iTRAQ8plex (Y)’; Peptide mass tolerance, ‘±100 p.p.m.’; Fragment mass tolerance, ‘±0.25 Da’; and Max missed cleavages, ‘1’. The Mascot search engine detected 215 proteins with a Mascot score above 45 from 10 244 MS/MS spectra.

## Results

### Proteins secreted from 3T3‐L1 cells during adipocyte differentiation

Mouse adipocyte 3T3‐L1 cells were cultured to clarify the types of proteins secreted during differentiation. Morphological change was observed in 3T3‐L1 cells following differentiation. Accumulation of intracellular lipid droplets, a typical differentiation marker, was found at D4. The size and the number of lipid droplets were increased at D8. To avoid contamination of serum proteins in DM1 and DM2, serum‐free media were used for the collection of secreted proteins. DM1 or DM2 were shifted to ITS^™^ premix media (ITS) that contained insulin, transferrin, and sodium selenite in DMEM, and cells were subsequently cultured for 2 days. Differentiated adipogenic cells were observed to morphologically maintain their maturation status in the presence of ITS (Fig. 1).

**Figure 2:**
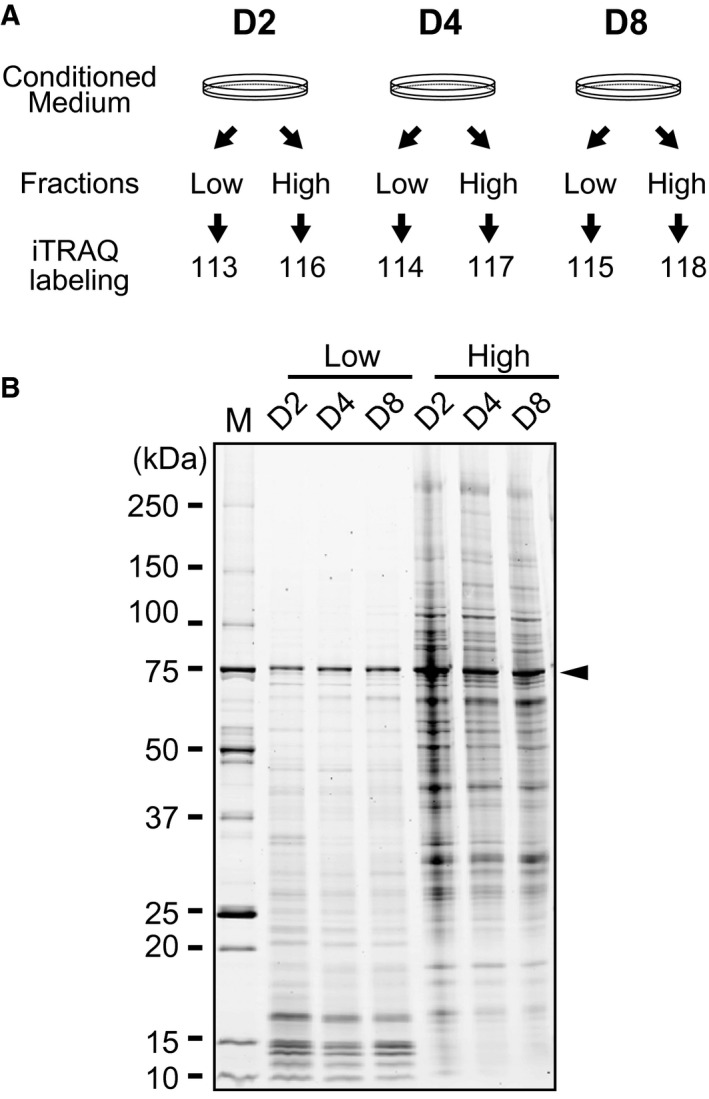
Preparation of secreted proteins. (A) A scheme of the experimental design comprising sample preparation and iTRAQ labeling. CM was collected and fractionated with a series of cut‐off spin columns for the removal of transferrin (76 kDa) derived from the ITS ^™^ supplement. ‘Low’ and ‘high’ fractions were fractionated using a series of spin columns. Each fraction of CM was labeled with a different iTRAQ ^®^ reporter as indicated. (B) Evaluation of secreted proteins by SDS/PAGE. Equivalent amounts of secreted proteins from each sample (D2, D4, and D8) were subjected to SDS/PAGE and subsequently stained with SYPRO ^®^ Ruby. The arrowhead indicates bands corresponding to transferrin. D2, day 2; D4, day 4; D8, day 8; M, molecular weight marker.

CM was collected and the quality of each was assessed by SDS/PAGE. Intense bands of approximately 76 kDa were observed on the gel and corresponded to transferrin that originated from the ITS supplement. To remove these proteins, CM was fractionated and concentrated using a series of spin columns followed by categorization into two fractions: ‘high’, which comprised a high molecular weight protein fraction, and ‘low’, which comprised a low molecular weight protein fraction (Fig. 2A). Fractionated specimens were subjected to SDS/PAGE (Fig. 2B). Although transferrin was not completely eliminated, the amount of contaminating transferrin was significantly reduced.

### Identification of secreted proteins by MS/MS analysis

**Figure 3:**
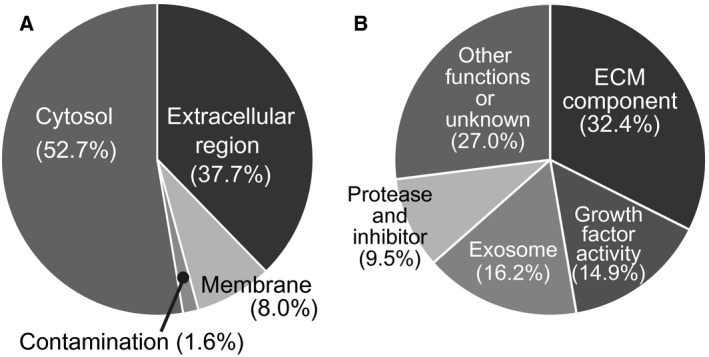
Subcellular localizations and functions of identified secreted proteins. (A) Identified proteins were categorized by subcellular localizations based on gene ontology. Secreted proteins were categorized in the extracellular region. Note that some proteins have multiple cellular localizations. (B) Secreted proteins in the extracellular region were further classified into five groups according to their functions: ECM component, growth factor activity, exosome, protease inhibitor, and others including unknown functions.

To characterize proteins secreted by 3T3‐L1 cells during adipocyte differentiation, the fractionated and concentrated CM was labeled with iTRAQ^®^ following trypsin digestion. Two‐dimensional peptide fractionation was performed and followed by the analysis of the fractionated specimens with a mass spectrometer. A total of 215 proteins were identified from 10 244 MS/MS spectra using the Mascot search engine. Identified proteins were categorized by their original localizations using gene ontology (Fig. 3A). Approximately 38% of identified proteins were derived from extracellular regions, 53% were cytoplasmic origin, 8% were membrane‐associated proteins, and 2% were from serum contamination.

Proteins localized to the extracellular region were further categorized into five groups by their functions. The number of identified proteins in the categories of ECM component, growth factor activity, exosome, protease inhibitor, and others including unknown functions were 32%, 15%, 16%, 10%, and 27%, respectively (Fig. 3B).

**Figure 4:**
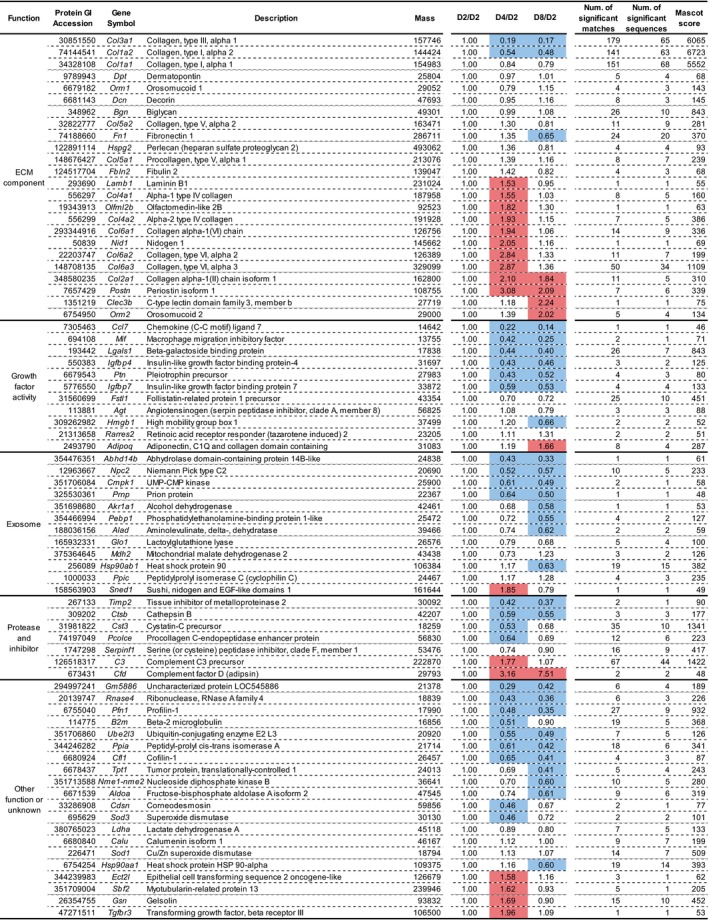
A list of secreted proteins categorized by functions. iTRAQ ^®^ signal intensities of identified proteins at each point of adipocyte differentiation were normalized with iTRAQ ^®^ signal intensities at D2. Intensities of iTRAQ 113 and iTRAQ 116 signals were used for normalization of low molecular weight proteins (< 60 kDa) and high molecular weight proteins (> 90 kDa), respectively. Proteins that altered greater than 1.5‐fold during adipocyte differentiation are shown in red rows. Proteins that altered less than 0.67‐fold during adipocyte differentiation are shown in blue rows. D2, day 2; D4, day 4; D8, day 8; Num., number.

To evaluate the relative expression levels of secreted proteins during adipocyte differentiation, signal intensities of the identified proteins were normalized with iTRAQ^®^ reporter signal intensities of D2 with 113 iTRAQ signal intensities for ‘low’ and 116 iTRAQ signal intensities for ‘high’. Identified proteins whose molecular weights were greater than 90 kDa or less than 60 kDa are depicted in Fig. 4. iTRAQ^®^ data showed four main patterns of secreted protein profiles. The first pattern demonstrated a gradual decrease in secreted levels during differentiation. The second pattern peaked at D4. The third pattern showed a gradual increment of secreted levels during differentiation. The last pattern demonstrated almost constant secretion levels during differentiation. These results indicate that 3T3‐L1 cells secrete distinct levels of various proteins during adipocyte differentiation.

## Discussion

### Identification of secreted proteins in the ECM component: collagen

Our iTRAQ data show that ECM components are the most abundant secreted proteins in differentiating adipocytes. Collagen, a major component of the ECM, comprises a 28 member superfamily of triple helical molecules in vertebrates 24. In the present study, we detected α‐subunits of type I, II, III, IV, V, and VI collagens, and found that each subunit of the collagen types was secreted at specific time points, namely, their secreted levels differ during adipocyte differentiation. A greater reduction in the secretion of α‐subunits of type I and type III collagens during adipocyte differentiation was observed. This releasing pattern may explain the construction of the extracellular fundamental structure that is composed of type I and type III fibril‐forming collagens at the early stage of adipocyte differentiation, as type I and type III collagens are classified as fibril‐forming collagens to provide extracellular scaffolds for cell attachment 24, 25. Intriguingly, secretion levels of type I and type III collagens were parallel to that of procollagen C‐endopeptidase enhancer protein that drives the enzymatic cleavage of procollagens to yield mature forms of triple helical collagen fibrils 26. This suggests that secreted α‐subunits of type I and type III collagens are possibly processed by the procollagen C‐endopeptidase enhancer protein for the maturation of collagen to form fibrils.

In contrast to type I and type III collagens, secretion of type IV, type V, and type VI collagen subunits peaked during the middle stage of differentiation. Network‐forming type IV collagen is one of the main components of the basement membrane (see below). Type V and type VI collagens are categorized as fibril‐forming collagen and beaded filament‐forming collagen, respectively. These collagens are associated with other type of collagens or ECM components. For example, type V and type VI collagens modify collagen fibrils that are composed of type I and type III collagens 25. Furthermore, type V collagen is closely associated with the basement membrane 27. Collagen VI alpha 3 (Col6a3) has been identified as one of the major adipocyte‐derived secretory proteins 9, 28. Collagen VI‐null mice demonstrated weakening of the extracellular scaffold of adipocytes 29. Therefore, our results may indicate that type V and type VI collagens modify collagen fibrils that consist of type I and type III collagens to support extracellular architecture during the middle stage of adipocyte differentiation.

Our results regarding the secreted collagen types and their expression pattern are largely consistent with other reports that have shown mRNA and protein expression levels of collagen types during adipocyte differentiation 11, 12, 15. Surprisingly, cartilage‐specific α‐subunits of type II collagen were detected in our iTRAQ data set. Mori and colleagues 13 also demonstrated type II collagen mRNA expression in subcutaneous adipose tissue by microarray analysis. Collectively, type II collagen may play a role in adipocytes and adipose tissues, although the specific role remains unclear.

### Identification of secreted proteins in the ECM component: basement membrane

In adipose tissues, each adipocyte is encircled by the basement membrane 30. Typical components of the basement membrane such as heparan sulfate proteoglycan 2, laminin β1 subunit, nidogen, and α‐subunits of type IV collagen were detected in our iTRAQ data set. These basement membrane components attained their peak during the middle stage of adipocyte differentiation upon observation of small lipid droplets. These results imply that basement membrane components are synchronously released from differentiating adipocytes to efficiently construct the basement membrane in conjunction with the accumulation of intracellular lipid droplets and to provide tensile strength to maturing adipocytes.

### Identification of secreted proteins in the ECM component: small proteoglycans

Decorin and biglycan, both members of the small leucine‐rich proteoglycan family, interact with type I collagen to organize collagen fibril formation and stabilization 31, 32, 33. Dermatopontin, a small ECM component, interacts with decorin 34, 35 or biglycan 35 to modify type I collagen‐based matrix assembly to provide scaffolds for cells. Our iTRAQ profile analysis revealed the release of relatively constant levels of these three proteoglycans during adipocyte differentiation. These proteoglycans may play a role in modifying type I collagen fibril‐based extracellular structural architecture, particularly during the early stage of adipocyte differentiation, when the secreted level of type I collagen subunits reaches its highest. Besides its structural function, small proteoglycans, particularly decorin, have been shown to act as a signaling molecule. We have previously shown that decorin promotes muscle differentiation via the activation of Akt downstream of insulin‐like growth factor I receptor 36. In adipocytes, an isoform of decorin plays a role as a functional receptor of resistin, a hormone that potentially links obesity to type II diabetes 37. Altogether, small proteoglycans may potentially have a dual role function, namely, as a modifying structural component and a signaling molecule depending on the stage of adipocyte differentiation.

### Growth factors

Adipokines such as adiponectin 4, 38, retinoic acid receptor responder 39, and complement C3 or adipsin 40 were detected in our iTRAQ data set. These adipokines were increasingly secreted during adipocyte differentiation and the highest levels of secretion was attained during late differentiation. This result indicates that adipocytes are more sufficiently secreted by maturing adipocytes than by young differentiating adipocytes. Although the iTRAQ analysis captured data on adipokines, the number of identified adipokines was low. This may be partially explained by the length of cell culture. Although 3T3‐L1 cells were cultured for 8 days following differentiation, the length of cell culture may have been insufficient to fully differentiate and mature to produce adipokines. Alternatively, the small number of identified adipokines may be caused by the low amounts of proteins used for iTRAQ labeling.

Secreted levels of adipocyte differentiation factors including pleiotrophin 41, macrophage migration inhibitory factor 42, and follistatin‐related protein 1‐like were decreased during adipocyte differentiation. This secretion pattern is supported by other studies that implicated these factors in the differentiation of adipocytes. Our observation of secreted levels of follistatin‐related protein 1‐like was consistent with a previous study that reported the downregulated expression of follistatin‐related protein 1‐like to be a hallmark of preadipocyte to adipocyte conversion 43.

Growth factors associated in promoting angiogenesis were frequently detected in our iTRAQ data set. These vascularization factors were follistatin‐related protein 1 44, pleiotrophin 45, high mobility group box 1 46, and insulin‐like growth factor binding protein‐4 and ‐7 47. Secretion levels were observed to be gradually decreased during adipocyte differentiation. This trend may indicate that juvenile adipocytes may more frequently secrete angiogenetic factors to facilitate vascularization than relatively mature adipocytes.

### Exosomes

Exosomes carrying functional microRNA and proteins are shed from cells as small vesicles to shuttle between cells 48. Increasing attention has been recently paid to exosomes as a mediator of intercellular communication 49. On the basis of the gene ontology classification in this study, approximately 16% of extracellular located proteins were classified as exosomal proteins (Fig. 3B). Our data were further verified using ExoCarta, a web‐based database for the analysis of exosome proteins [http://www.exocarta.org/ (release date, 29 July 2015)] 50. Accordingly, approximately 75% of total identified proteins were classified as exosomal proteins by the ExoCarta database. These results imply that some proteins which are categorized as cytoplasmic proteins by the gene ontology classification are secreted as components of exosomes although some proteins may be released from cytosol of dead cells. Adipocytes have been assumed to secrete functional exosomes 51, 52 and adipocyte‐derived exosomes contain mediators capable of activating end‐organ inflammatory and fibrotic signaling pathways 53. In consideration of these assumptions, differentiating adipocytes may more frequently shed exosomes as a mediator of extracellular communication than typical growth factors that contain classical secretion signal peptides.

### Protease inhibitors

Tissue inhibitor of metalloproteinase 2 (Timp2) was detected in our iTRAQ data set, and decreased during adipocyte differentiation. This trend is consistent with type I and type III collagens. As Timp2 inhibits the breakdown of ECM structures by matrix metalloproteinases, adipocytes facilitate the production of ECM components to assemble ECM structures by secreting collagens and Timp2 at the early stage of adipocyte differentiation.

Cathepsin B, a member of the cysteine protease superfamily, is generally thought to be located in intracellular lysosomes; however, cathepsin B has been detected in secreted fractions. The observation of the decreased secretion of cathepsin B during adipocyte differentiation in our study may reflect the function of cathepsin B in adipocyte differentiation, as extracellular cathepsin promotes adipocyte differentiation possibly through ECM degradation 54.

## Conclusions

**Figure 5:**
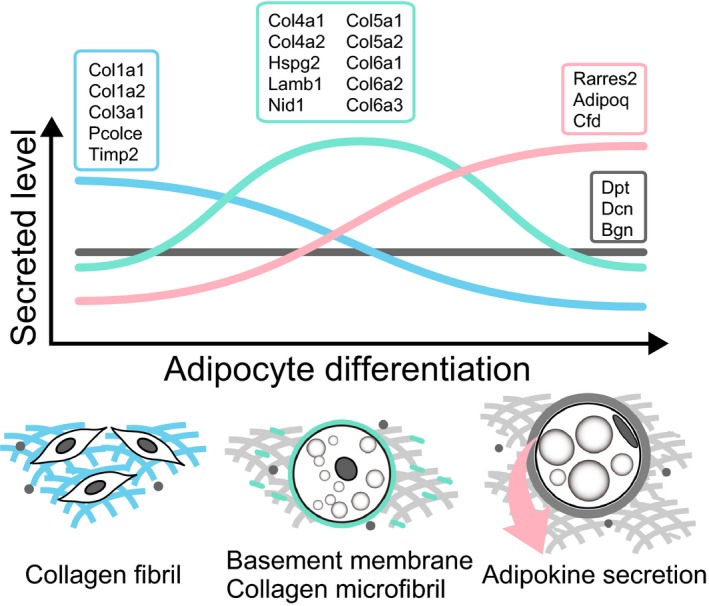
Schematic diagram of 3T3‐L1 cell‐secreted proteins during adipocyte differentiation. During the early stage of adipocyte differentiation, 3T3‐L1 cells secreted fundamental components of the ECM to build cellular scaffolds such as subunits of type I (Col1a1 and Col1a2) and type III (Col3a1) collagens in conjunction with collagen associated proteins, e.g., procollagen C‐endopeptidase enhancer (Pcolce) and metallopeptidase inhibitor 2 (Timp2). The basement membrane components including perlecan (Hspg2), laminin B1 (Lamb1), nidogen 1 (Nid1), and subunits of collagen type IV (Col4a1, Col4a2) peaked at the middle stage of adipocyte differentiation. Subunits of microfibrillar collagen components such as type V (Col5a1, Col5a2) and type VI (Col6a1, Col6a2, Col6a3) collagens also attained their greatest levels. During the late stage of adipocyte differentiation, 3T3‐L1 cells facilitated to secrete adipokines, such as retinoic acid receptor responder 2 (Rarres2), adiponectin (Adipoq), and adipsin (Cfd), rather than extracellular components. Small proteoglycan components, such as dermatopontin (Dpt), decorin (Dcn), and biglycan (Bgn), were secreted at relatively constant levels throughout adipocyte differentiation. 3T3‐L1 cells secreted distinct types and amount of proteins at different stages of adipocyte differentiation.

The quantitative proteomic study reported here provides an overview of secreted protein profiles in differentiating adipocytes (Fig. 5). A considerable variety of ECM components and growth factors including adipokines were detected in the proteomic data sets. During the early stages of adipocyte differentiation, secretion of fundamental ECM components such as type I and type III collagens were at the highest levels, but levels gradually reduced during differentiation. During the middle stage of adipocyte differentiation, basement membrane components and their associated proteins reached their peak levels. As adipocyte differentiation progressed, adipokine secretion levels were increased, although levels of most growth factors decreased. Our data demonstrate that differentiating adipocytes regulate the release of types, amount, and timing of proteins for the construction of an optimal extracellular microenvironment for differentiating adipocytes.

## Author contribution

KO and TN conceived and designed the project. KO and IN acquired data. KO, MO, SM, and TN analyzed and interpreted the data. KO wrote the paper.
